# Specific electrochemical iodination of horse heart myoglobin at tyrosine 103 as determined by Fourier transform ion cyclotron resonance mass spectrometry

**DOI:** 10.1016/j.abb.2008.02.032

**Published:** 2008-06-01

**Authors:** Jesus Iniesta, Helen J. Cooper, Alan G. Marshall, John Heptinstall, David J. Walton, Ian R. Peterson

**Affiliations:** a Centre for Molecular and Biomedical Science, Faculty of Health and Life Sciences, Coventry University, Coventry CV1 5FB, UK; b Ion Cyclotron Resonance Program, National High Magnetic Field Laboratory, Florida State University, Tallahassee, FL 32310, USA; c Department of Chemistry and Biochemistry, Florida State University, Tallahassee, FL 32306, USA

**Keywords:** Iodotyrosine, Electrooxidation, Protein modification, Iodination, Horse heart myoglobin, Mass spectrometry, FT-ICR, FT-MS, ECD, IRMPD

## Abstract

The iodination of proteins remains a useful tool in biochemistry for radiolabelling. However, chemical or enzymatic iodination is difficult to control and can give deleterious polyiodination. Previously, we have shown that electrooxidation with nitrite is a rapid method for the selective nitration of tyrosine residues in proteins. In principle, it should be possible to substitute a number of electrooxidisable anions into the tyrosine phenol ring.

Electrochemical iodination is more difficult to control than nitration because the rapid anodic oxidation of I^−^ leads to persistent formation of the iodinating triiodide anion. However, application of pulsed electrooxidation and reduction cycles is shown to be an effective procedure for the selective mono and double-iodination of myoglobin, which may have general application to other proteins in controlling of the level of iodination.

Mono- and double-iodination of myoglobin by this method was confirmed by electrospray FT-ICR mass spectrometry. Infrared multiphoton dissociation (IRMPD) enabled localization of the site of mono-iodination to be restricted to either His97 or Tyr103. More extensive sequence coverage was obtained with electron capture dissociation (ECD), allowing unambiguous assignment of the site of iodination to Tyr103.

Iodination of proteins is an important tool in radioimmunoassays (e.g., [1]), receptor binding [2], determination of protein structure [3], production of radioactive peptide imaging agents [4] and generally as a marker for proteins [5,6]. Iodination is a chemical or enzymatic process usually involving the triiodide ion or iodide ion, together with an oxidizing agent such as chloramine-T or hydrogen peroxide plus peroxidase, followed by quenching with a reducing agent. More recently iodo-gen (1,3,4,6-tetrachloro-3a,6a-diphenyl-glycoluril) [7,8] has found increasing use since it offers a measure of control when used as a coating on the walls of reaction vessels followed by subsequent decanting of the iodinated protein to terminate the reaction. Mono- and diiodination of tyrosyl residues together with iodohistidyl residues are the principal modifications in chemical and enzymatic modification but, in general, these methods are difficult to control and can give deleterious polyiodination. Moreover, iodine can cause oxidation of sulphydryl groups and tryptophan.

Electrosynthetic modification of proteins is a new method for production of novel functionalities at specific amino acid residues including methionine, trytophan [9] and tyrosine [10]. We have described the selective electronitration of horse heart myoglobin [11] and hen egg white lysozyme (HEWL)^3^ [12] at specific tyrosine residues in the presence of nitrite. The reaction can be controlled by the applied potential to yield mono and bis nitration in lysozyme, in contrast to chemical nitration with tetranitromethane [13] or peroxynitrite [14] in which combinations of mono, bis, and tris nitration are observed.

Since the mechanism of electronitration involves the anodic oxidation of nitrite, it is likely that other anions, with oxidation potentials less than that causing protein damage, could be substituted for nitrite in the electrooxidation. The electrooxidation of iodide in protein iodination has been previously published as a patented method [15], involving a potential of 600–650 mV (vs Ag/AgCl) in a divided cell with an anode preadsorbed with nonradiaoactive iodide to act as carrier for the radiolabel. To obtain a high yield of radioiodinated protein, a molar ratio of protein:iodide of 5:1 was recommended, but the products were not fully analyzed. A similar approach was taken in the electrochemical preparation of radioactive iodinated parathyroid hormone [16]. In this case a molar ratio of 1:1, protein: iodide, both in the nanomolar range, was used in order to give a very high specific radioactivity and to favor monoiodination of tyrosine, since monoiodotyrosine is a more avid acceptor of iodine than is tyrosine itself [17]. Electrochemical conditions were a constant current of 40 μA leading to a maximum allowed potential of +750 mV (vs SCE).

In this paper, we present a new electrochemical procedure for the selective mono and diiodination of horse heart myoglobin (Mb), in which the iodide concentration can be elevated, leading to a specific and selective tyrosyl iodination at residue Y103. Specific mono- and double-iodination of the protein was confirmed by use of electrospray Fourier transform ion cyclotron resonance (FT-ICR) mass spectrometry [18]. FT-ICR offers ultrahigh resolution and mass accuracy and is ideally suited to the top–down analysis of proteins and characterization of their modifications. The modified proteins were subjected to infrared multiphoton dissociation (IRMPD) [19] and electron capture dissociation (ECD) [20] in order to identify the site(s) of modification. The results show that the method described results in iodination at Y103.

## Materials and methods

### Materials

Horse heart myoglobin was purchased from Sigma–Aldrich Chemical Co. and was used as received. All other reagents were the highest grade commercially available and were used without any purification. Solutions were prepared with ultrapure water, 18 mΩ cm (ELGA maxima System).

### Electrochemical iodination of horse heart myoglobin

Cyclic voltammetry for the study of electrooxidation of iodide was performed in a one-compartment cell of 50 mL with a reference compartment attached via a Luggin capillary. Experiments were performed with 20 or 1 mM KI in aqueous solution with 50 mM disodium tetraborate and 0.5 M sodium sulfate as supporting electrolyte adjusted to a final pH of 9.0 by use of boric acid. A platinum wire (0.5 mm diameter, 0.19 cm^2^ immersed) was used as working electrode for the voltammetric studies. The counter electrode was a spiral wound platinum wire and the reference electrode was a saturated calomel electrode. Both the working and the counter electrodes were treated in a hot flame for a few seconds, then cooled in air and finally rinsed with ultrapure water. Solutions were deaerated with argon. A small stream of argon was maintained during the cyclic voltammetry experiments which were carried out with an Autolab PGSTATS 30 apparatus, at 298 ± 2 K with a scan rate of 0.05 V s^−1^.

### Preparative electrolyses

Preparative electrolysis was performed with an EG& G Model 273 computerized potentiostat/galvanostat by use of 270–250 Research Electrochemistry Software for voltammetric data processing. In a typical preparative electrolysis, an H-cell with compartments divided by sintered glass was charged in each compartment with 50 mL of 50 mM disodium tetraborate, pH 9.0, adjusted with boric acid. Horse heart myoglobin (25 mg, to give 0.5 mg mL^−1^) was added to the anodic compartment. Solutions were stirred magnetically and placed on ice. Solutions were not deoxygenated. The working electrode (anode) was a 60 cm^2^ geometric area platinum mesh basket and the counter electrode was a 4 cm^2^ platinum flag, with saturated calomel as the reference electrode. The platinum mesh basket electrode was cleaned with an aqueous solution containing detergent (Decon 90) for 20 min in an ultrasonic cleaning bath (180 W, 40 kHz). Thereafter, the electrode was thoroughly rinsed with water. Two distinctly different procedures (methods A and B, respectively) were performed for the electrochemical iodination of myoglobin.

In method A, electrolysis was carried out with 20 mM KI added to the anodic compartment and the potential was set at 0.4 V for 1, 5, or 10 min, respectively. A total charge of 690, 2610, or 4890 mC was passed through the system, corresponding to the production of 3.0, 15.8, or 21.5 mol of iodine per mol of myoglobin. In method A, excess iodine was reduced by adding 0.2 mL of 1 M Na_2_S_2_O_3_ solution.

In method B, 1 mM KI was used in the anodic compartment together with 6 mM sodium sulfate to maintain the ionic strength as in method A, and a cyclic pulsed potential was applied, whereby potentials were set at 0.4 V for 2.5 s followed by 0 V for 5 s. The electrolysis times were 30 or 52.5 min, respectively (240 or 420 cycles), which means that the working electrode was held at 0.4 V for a cumulative time of 10 or 17.5 min, respectively.

Iodinated myoglobin samples were dialyzed against 10 mM NH_4_HCO_3_ at pH 8.0 by use of a dialysis membrane with a 3.5 kDa cut off (Spectra/Por^™^ number 3 regenerated cellulose dialysis membranes, from SPECTRUMLABS (Espectrum Europe, P.D. Box 3262, 4800 DG Breda, The Netherlands) and lyophilized.

### Ultraviolet/visible spectra (UV–vis)

Absorption spectra were collected with a UNICAM UV–vis Type UV4 spectrophotometer.

### Mass spectrometry

Solutions of iodinated myoglobin samples were prepared by addition of methanol:water (1:1) (J.T. Baker, Philipsburg, NJ), 1% acetic acid (Aldrich, Milwaukee, WI) to the lyophilized samples for a final concentration of 1 μM. The samples were analyzed with a custom-built, passively-shielded, 9.4 T FT-ICR mass spectrometer equipped with an external microelectrospray ionization source [21]. The samples were infused at a flow rate of 300 nL/min through an electrospray emitter consisting of a 50 μm i.d. fused silica capillary which had been mechanically ground to a uniform thin-walled tip [22]. 2.0 kV was applied between the microelectrospray emitter and the capillary entrance. The ions were externally accumulated for 2–5 s in an rf-only octapole. The ions were transferred through multipole ion guides and trapped in an open [23] cylindrical cell (Malmberg-Penning trap) [24].

Ions were frequency-sweep (“chirp”) excited (72–640 kHz, at 150 Hz/μs) and detected in direct mode (512 kword time-domain data). Twenty time-domain data sets were co-added, Hanning apodized, zero-filled once and subjected to fast Fourier transform (FFT) followed by magnitude calculation. The experimental event sequence was controlled by a modular ICR data acquisition system (MIDAS) [25]. The FT-ICR mass spectra were internally frequency-to-*m*/*z* calibrated [26] with respect to the unmodified myoglobin ions. The FT-ICR mass spectra were analyzed by use of the MIDAS analysis software package [27].

### Electron capture dissociation and infrared multiphoton dissociation

For tandem mass spectrometry, the precursor ions were externally accumulated for 4–5 s in an rf-only octapole. A front-end resolving quadrupole and/or stored-waveform inverse Fourier transform (SWIFT) [28,29] ejection served to isolate the peptide ions of interest. An indirectly heated dispenser cathode (Heat Wave, Watsonville, CA) mounted on the central axis of the system provided the electrons for ECD. A potential of −1.5 V was applied to the cathode during the irradiation event. A grid situated in front of the filament was kept at −200 V for most of the experiment and pulsed to +200 V during the ECD event. The isolated precursor ions were irradiated with electrons and photons for 10 ms. Two-hundred and fifty time-domain data sets were co-added, Hanning apodized, zero-filled once and subjected to fast Fourier transform (FFT) followed by magnitude calculation. The ECD mass spectra were internally frequency-to-*m*/*z* calibrated with respect to the precursor ion and the charge reduced species or backbone fragments.

An off-axis continuous wave 40 W, 10.6 μm wavelength CO_2_ laser (Synrad E48-2-115, Bothell, WA) fitted with a beam expander provided the photons for infrared multiphoton dissociation (IRMPD). The isolated parent ions were irradiated with photons for 1 s. Twenty time-domain data sets were co-added, Hanning apodized, zero-filled once and subjected to fast Fourier transform (FFT) followed by magnitude calculation. The IRMPD mass spectra were internally frequency-to-*m*/*z* calibrated with respect to the precursor ion and backbone fragments.

## Results and discussion

**Fig. 1:**
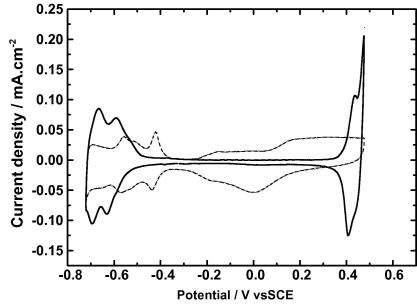
Cyclic voltammogram of potassium iodide at a platinum anode. Cyclic voltammetry for the electrooxidation of iodide was performed in a one-compartment 50 mL cell with a reference compartment attached via a Luggin capillary. Experiments were performed with 1 mM KI in aqueous solution with 50 mM disodium tetraborate as supporting electrolyte adjusted to pH 9.0 with boric acid. A polycrystalline platinum electrode with a geometric area of 0.149 cm^2^ was used as working electrode, with a spiral wound platinum wire counter electrode and a saturated calomel reference electrode. Solutions were deaerated with argon and a stream of argon was maintained during cyclic voltammetry at 298 ± 2 K with a scan rate of 0.05 V s^−1^. (–·–·–, Background, 5th scan; ——, +KI, 5th scan).

Cyclic voltammetry of 20 mM KI demonstrated that aqueous iodide is electrochemically oxidized to aqueous iodine (rapid brown coloration) at a potential of 0.4 V vs SCE (Fig. 1); therefore that value was the potential fixed for the electrochemical iodination. Other peaks in the cyclic voltammogram (CV) are assigned as, in the background CV: anodic wave between +0.1 and +0.47 V, oxidation of platinum; cathodic peak at 0.0 V seen in the reverse as reduction of platinum oxide; aniodic and cathodic peaks between −0.3 and −0.72 V, adsorption and desorption of hydrogen and anions (tetraborate and borate) with hydrogen evolution beginning at −0.71 V. In the KI-containing CV; peaks between −0.5 and −.072 V, adsorption and desorption of hydrogen and anions, the potential shift being due to adsorption of iodine on the platinum surface.

**Fig. 2:**
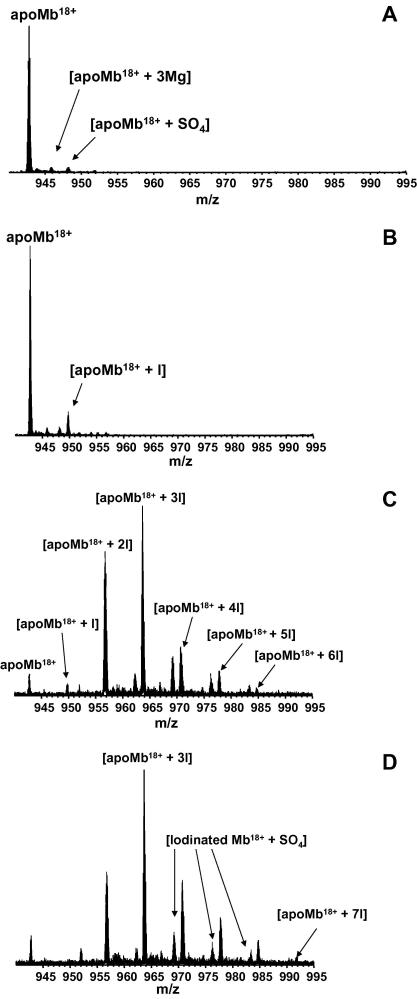
Expanded *m*/*z* region showing the 18+ charge state in the ESI FT-ICR mass spectra of horse heart myoglobin. (A) Mb (1 μM) in water/methanol (1/1, v/v, 1% acetic acid). (B) Iodinated Mb (1 μM) in water/methanol (1/1, v/v, 1% acetic acid), electrochemical iodination at 0.4 V vs SCE for 1 min, 20 mM KI, (C) Iodinated Mb (1 μM) in water/methanol (1/1, v/v, 1% acetic acid), electrochemical iodination at 0.4 V vs SCE for 5 min, 20 mM KI, (C) Iodinated holoMb (20 μM) in water/methanol (1/1, v/v, 1% acetic acid), electrochemical iodination at 0.4 V vs SCE for 10 min, 20 mM KI.

The electrochemical iodination of horse heart myoglobin was performed initially with method A, i.e., 20 mM KI in a buffered solution (50 mM disodium tetraborate adjusted to pH 9.0) for 1, 5, and 10 min of electrolysis. Fig. 2A shows the expanded *m*/*z* region showing the 18+ charge state of the electrospray ionization (ESI) FT-ICR mass spectrum of untreated horse heart apomyoglobin (1 μM) obtained in water/methanol (1/1, v/v, 1% acetic acid). (Note, holomyoglobin is denatured in the organic solvent and the heme group is released). Fig. 2B shows the expanded *m*/*z* region showing the 18+ charge state of the ESI FT-ICR mass spectrum for the iodinated Mb after 1 min treatment. Peaks corresponding to apoMb and singly-iodinated apoMb were observed. The mass difference between the two species was 125.9 Da, corresponding to [+I − H]. Although the electrochemical iodination of Mb for 1 min under the above conditions results in a single iodination, the yield is poor. Fig. 2C shows the ESI FT-ICR mass spectrum of the most abundant charge state, 18+, obtained for the iodinated holoMb after 5 min treatment. The extent of iodination of Mb increased markedly, but treatment resulted in multiple iodinated species and a brown coloration observed during electrolysis. Fig. 2D shows the ESI FT-ICR mass spectrum of iodinated holoMb obtained after 10 min of electrochemical treatment. The spectrum shows up to 7 iodine atoms incorporated into Mb.

Iodide ions may undergo spontaneous oxidation (absorbate charge transfer) to atomic iodine on platinum at a range of potentials between 0 and 0.4 V vs SCE [30]; moreover molecular iodine will be formed electrochemically at higher potentials leading to soluble or adsorbed molecular iodine on the electrode surface. The iodination mechanism (halogenation in general) is unclear and it may involve either homogeneous iodination by anodically formed atomic or molecular iodine, or nucleophilic iodide attack of the tyrosinate radical. The formation of tyrosinate radicals in anodically oxidized Mb has not been proved, but electrochemical iodination might also occur in a coupling reaction of both atomic iodine and tyrosinate radical Mb. Such a situation would be advantageous in terms of selectivity and control of the process on the electrode surface. The electrochemical iodination of Mb in 20 mM KI for 1 min involves a final ratio of ∼3 mole I_2_ electrogenerated/mole Mb. In this way iodination may be mostly achieved near/upon the electrode surface and, therefore, side reactions or uncontrollable iodination are lessened at short iodination times but the yield is low.

Moreover, at longer electrolysis times (5 or 10 min in 20 mM KI) there is the formation of triiodide from the reaction between aqueous iodide and electrochemically formed aqueous iodine, in equilibrium with solid iodine. The triiodide subsequently diffuses into the bulk solution. Therefore iodination will mainly occur in the bulk solution by aqueous triiodide. Thus multiple polyiodinated Mb species are observed, as shown for the electrochemical iodination for 5 and 10 min (see Fig. 2C and D).

**Fig. 3:**
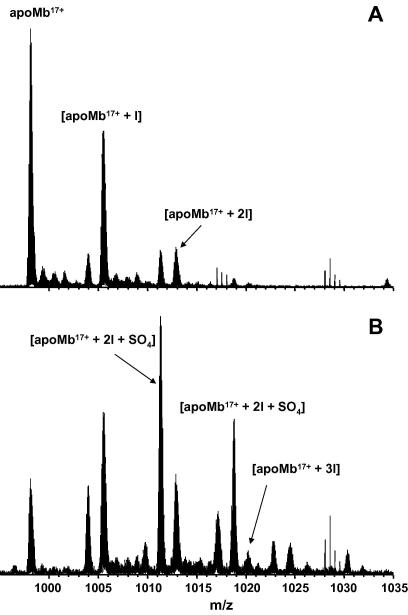
Expanded *m*/*z* region showing the 17+ charge state in the ESI FT-ICR mass spectra of horse heart myoglobin. (A) Iodinated Mb (1 μM) in water/methanol (1/1, v/v, 1% acetic acid), electrochemical iodination with cyclic pulsed potentials (2.5 s at 0.4 V vs SCE and 5 s at 0.0 V vs SCE, 240 cycles,), (B) Iodinated Mb (1 μM) in water/methanol (1/1, v/v, 1% acetic acid), electrochemical iodination with cyclic pulsed potentials (2.5 s at 0.4 V vs SCE and 5 s at 0.0 V vs SCE, 420 cycles).

In Method B a cyclic pulsed potential between 0 and 0.4 V was performed for the electrochemical iodination of Mb in order to briefly form iodine and reduce it back to iodide in each cycle to improve the selectivity and specificity for iodination. Potentials were set at 0.4 V for 2.5 s and 0 V for 5 s. Net charge passed was nearly zero for the whole process. Fig. 3A shows the expanded *m*/*z* region showing the 17+ charge state of the ESI FT-ICR mass spectrum of the iodinated holoMb based on the cyclic pulsed potentials (240 cycles, 10 min at 0.4 V vs SCE). Charge states 12+ through 22+ were observed. The iodination process was attenuated and peaks corresponding to singly- and doubly-iodinated Mb (Δ_mass_ = 125.9 and 251.8 Da, respectively) were observed. No peaks corresponding to triiodinated species were observed. Fig. 3B shows the same *m*/*z* region of the ESI FT-ICR mass spectrum obtained following 52.5 min electrolysis (420 cycles, 17.5 min at 0.4 V vs SCE).

As above, the electroiodination of Mb results mainly in singly- and doubly-iodinated species. A small peak corresponding to triply-iodinated apo-Mb was also observed. There was no evidence for higher iodinated species. Peaks containing sulfate ions were apparent in Figs. 2 and 3. Whilst 6 mM sodium sulfate was added in Method B which would explain these peaks (Fig. 3), no such addition was made in Method A (Fig. 2). It is probable that ubiquitous sulfate ions were incorporated during the purification and dialysis process.

We conclude that the low concentration of aqueous iodide (1 mM KI; molar ratio protein: iodide ∼1:30) and the pulsed cycles between 0 and 0.4 V vs SCE dramatically improve the selectivity of iodination and the yield of mono- and doubly-iodinated protein. Aqueous molecular iodine, *I*_2(aq)_, is anodically formed at 0.4 V for 2.5 s and thereafter it is reversibly electrochemically reduced to aqueous or adsorbed iodide when the potential is held at 0 V vs SCE for 5 s. In this way, electroiodination of Mb occurs on/near the electrode surface, avoiding the formation of aqueous iodine and triiodide in the bulk solution and eliminating the brown coloration during iodination. Moreover, there is no evidence for other covalent modifications of Mb produced either by direct oxidation of Mb on the electrode or chemical oxidation by triiodide ions.

The chemical iodination of phenols shows a marked dependence on pH with a 42× increase in rate between pH 6 and 8 [31]. Consideration of alternative iodinating species (I_2_, ${\text{I}}_{3}^{-}$, HOI, I^+^ or I), with either the phenol or phenolate anion as intermediates, led to the conclusion that the reacting species were I_2_ and the phenolate anion [31], although there was no attempt to specifically generate atomic iodine. That a similar mechanism may be operating electrochemically is indicated by a consideration of previous and the current work in this area. Thus electrooxidation will lead to I_2_(aq) production since iodide is easily oxidized to iodine yielding molecular iodine in solution at +0.358 V (vs SCE) in 0.1 M H2SO4 [30], (0.4 V at pH 9 in the current investigation) in accordance with reaction 1.

(1) $$2{\text{I}}^{-}(\text{aq})\;⇆\;{\text{I}}_{2}(\text{aq})$$

Moreover the accumulation of adsorbed iodine atoms [32] is neglected, consistent with the reversibility of the I^−^/I_2_ system [30]. The solubility of I_2_ in water is not exceeded (1.1 mM at 25 C, [33]) to give solid iodine since a concentration of I^−^ in excess of 3 mM is required in electrooxidation to bring this about [30], whereas it is 1 mM in the redox pulsed method: moreover in the latter the net charge passed is effectively zero therefore iodine is only transiently formed.

There is the possibility of transient formation of ${\text{I}}_{3}^{-}$ from reaction 2.

(2) $${\text{I}}_{2}(\text{aq})+{\text{I}}^{-}\;⇆\;{\text{I}}_{3}^{-}(\text{aq})$$

K_eq_ here is about 600 M^−1^ [30,32] and the forward rate constant is 1 × 10^5^ mol^−1^ cm^3^ s^−1^ [30]. Either I_2_(aq) or ${\text{I}}_{3}^{-}$ could be the iodinating species in the redox pulsed system. Chemical iodination of phenolate anions is faster than that of the undissociated phenol [31]. Since the pH of the redox pulsed system is 9.0, tyrosinate anions in myoglobin will be present. The undissociated phenol in tyrosine will not be oxidized at +0.4 V (vs SCE) since the potential for the oxidation of phenol with various substituents is given as +0.7 − 1.65 V (vs SCE) [34–36] whereas the phenolate anion is much more easily oxidized in a 1 e^−^ oxidation, with potentials in the range −0.5 − +0.12V (vs SCE) [34,35,37]. It is likely therefore that, at the operating potential of the redox pulsed system, neutral phenoxy radicals are generated at the anode in myoglobin and that iodination occurs, by aqueous I_2_ or ${\text{I}}_{3}^{-}$, of either the generated radical or of the tyrosinylate anion.

**Fig. 4:**
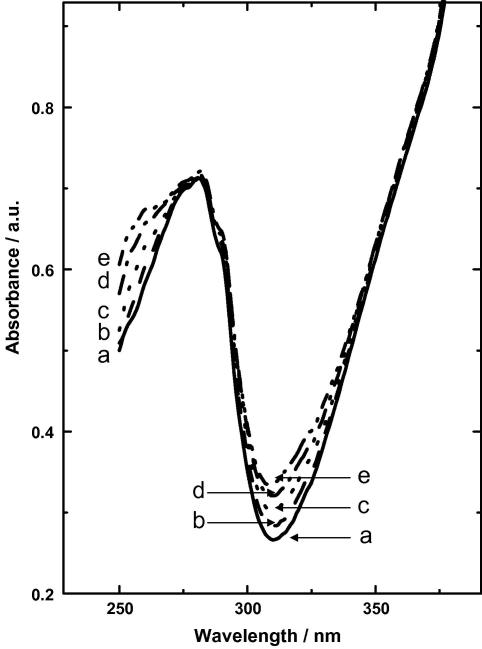
Spectral changes of myoglobin on iodination. Electrochemical iodination of 0.5 mg mL^−1^ of holomyoglobin using cyclic pulsed potential for an accumulative time of 10 min at 0.4 V (vs SCE). Time of electrolysis indicated (min): a, zero; b, 2.5; c, 5; d, 7.5; e, 10.

Mono- and diiodotyrosine have *λ*_max_ of 305 and 311 nm and molar extinction coefficients of 4100 and 6250, as phenolate anions, respectively [38]. The spectrum (Fig. 4) shows an increasing absorption at these wavelengths with time in electroiodination. Calculation from the 10 min exposure to 0.4 V, when the predominant product is monoiodomyoglobin, gives a concentration of 14.4 μM monoiodomyoglobin, a yield of 51%. That yield is consistent with that indicated in the corresponding mass spectrum (Fig. 3A).

**Fig. 5:**
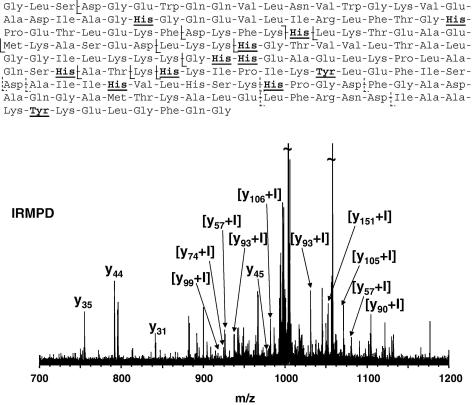
IRMPD fragmentation of singly-iodinated myoglobin. Bottom: IRMPD FT-ICR mass spectrum of [M + 17H]^17+^ ions of singly-iodinated myoglobin. Top: b and y fragments observed. Fragments denoted with dashed line were observed without addition of iodine. Electrochemical iodination with pulsed cyclic potentials (2.5 s at 0.4 V vs SCE and 5 s at 0.0 V vs SCE, 240 cycles).

Infrared multiphoton dissociation (IRMPD) and electron capture dissociation (ECD) fragmentation techniques were used to determine the site of iodination. IRMPD is a slow-heating tandem mass spectrometry technique that results in fragmentation of the precursor ion by the lowest energy pathway. For peptides and proteins, cleavage of the peptide bond results in b and y fragment ions in which the charge is retained on the N- and C-termini, respectively. Fig. 5 shows the IRMPD spectrum obtained from [M + 17H]^17+^ ions of iodinated Mb produced by the cyclic pulsed potential method together with a summary of the fragments observed. Myoglobin contains ten histidine residues and two tyrosine residues, each of which is a potential site of iodination. Fragments y_12_, y_17_, y_31_, y_35_, y_44_, y_45_, b_47_, b_54_ and b_62_ were observed without iodine modification. Fragments y_57_, y_58_, y_60_, y_74_, y_90_, y_93_, y_99_, y_105_, y_106_, y_110_, y_150_ and y_151_ were observed with a mass increase of 125.9 Da over the expected value. The IRMPD results thus suggest that iodination occurs either on His97 or Tyr103.

**Fig. 6:**
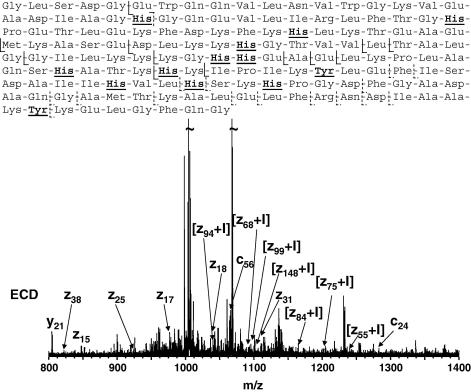
ECD fragmentation of singly-iodinated myoglobin. Bottom: ECD FT-ICR mass spectrum of [M + 17H]^17+^ ions of singly-iodinated myoglobin. Top: c and z fragments observed. Fragments denoted with dashed line were observed without addition of iodine. Electrochemical iodination with pulsed cyclic potentials (2.5 s at 0.4 V vs SCE and 5 s at 0.0 V vs SCE, 240 cycles).

ECD is a recently developed tandem mass spectrometry technique. ECD of peptides and proteins results in cleavage of the N—C*α* bond to produce c and z ions, in which the charge is retained on the N- and C-termini, respectively. Unlike IRMPD, ECD seems to be a non-ergodic technique, i.e., cleavage occurs before energy randomisation. Consequently, ECD is not site-specific and the extent of cleavage is greater providing higher sequence coverage. Fig. 6 shows the ECD spectrum obtained from [M+17H]^17+^ ions of iodinated Mb produced by the cyclic pulsed potential method together with a summary of the fragments observed. Fragments z_7_, z_12_, z_13_, z_14_, z_15_, z_17_, z_8_, z_21_, z_24_, z_25_, z_31_, z_37_, z_38_, z_47_, z_48_, and c_24_ were observed unmodified. Fragments z_55_, z_57_, z_68_, z_69_, z_70_, z_75_, z_80_, z_84_, z_85_, z_94_, z_99_, and z_128_ were observed with a mass shift of 125.9 Da. The site of mono-iodination can therefore be unambiguously assigned to Tyr103, which is also the site of electronitration of myoglobin [12]. It is most likely that the doubly-iodinated protein is bisiodo—at Tyr103, given the avidity of monoiodotyrosine for further iodination [17].

The redox pulse method can give control of iodination even at high KI concentrations when high specific activity radioactive iodine is not important. The method could also be applied to very low iodide concentrations to produce high specific activity iodoprotein, allowing for exquisite control of the iodination process.

## Footnotes

3 *Abbreviations used:* HEWL, hen egg white lysozyme; Mb, myoglobin; FT-ICR, Fourier transform ion cyclotron resonance; IRMPD, infrared multiphoton dissociation; ECD, electron capture dissociation; FFT, fast Fourier transform; MIDAS, modular ICR data acquisition system; SWIFT, stored-waveform inverse Fourier transform; CV, cyclic voltammogram; ESI, electrospray ionization.
